# LncRNA CACS15 regulates tongue squamous cell carcinoma cell behaviors and predicts survival

**DOI:** 10.1186/s12903-019-0924-0

**Published:** 2019-10-29

**Authors:** Xiao Wu, Jing Ma, Jian Chen, Han Huang

**Affiliations:** 1 Oral and Maxillofacial Surgery Ward, The first Affiliated Hospital of Jinzhou Medical University, No. 2, Section 5, Renmin Street, Jinzhou City, Liaoning Province 121000 People’s Republic of China; 2 Department of Stomatology, Huludao Second People’s Hospital, Huludao City, Liaoning Province 125000 People’s Republic of China

**Keywords:** Tongue squamous cell carcinoma, lncRNA CASC15, miR-124, Prognosis

## Abstract

**Background:**

The involvement of lncRNA CASC15 in several types of cancers has been reported, while its role in tongue squamous cell carcinoma (TSCC) is unknown. Our study aimed to investigate the clinical potentials of lncRNA CASC15 in TSCC.

**Methods:**

The expression of CASC15 and miR-124 in tissue samples from TSCC patients and TSCC cell lines was analyzed by qPCR. Overexpression experiments were performed to analyze the interactions between CASC15 and miR-124. Survival analysis was performed to analyze the prognostic values of CASC15 and miR-124 for TSCC. Transwell assays were performed to analyze cell invasion and migration.

**Results:**

We found that CASC15 was upregulated, while miR-124 was downregulated in TSCC tissues than in non-cancer tissues of TSCC patients. CASC15 and miR-125 expression was not significantly different among patients with different clinical stages, and patients with high level of CASC15 and low level of miR-125 showed low overall survival rate. CASC15 and miR-124 were inversely correlated in TSCC tissues, and CASC15 overexpression in TSCC cells resulted in downregulation of miR-124. In contrast, overexpression of miR-124 showed no significant effect on CASC15 expression. CASC15 overexpression resulted in the increased, while miR-124 overexpression resulted in the decreased migration and invasion rates of TSCC cells.

**Conclusion:**

CASC15 and miR-124 predict TSCC patients’ survival and CASC15 may downregulate miR-124 to inhibit TSCC cell migration and invasion.

The study was approved by Ethics Committee of The first Affiliated Hospital of Jinzhou Medical University (20103548FAHJMU).

## Background

Oral cancer accounts for 2% of all cancer cases and causes 1.9% of all cancer-related deaths [1]. In developing countries, oral cancer is the 3rd most common types of malignancy [1, 2]. The most common type of oral cancer is squamous cell carcinoma, which mainly affects tongue [3]. Tongue squamous cell carcinoma (TSCC) accounts for 25 to 40% of oral cancers and is leading cause of deaths among oral cancer patients due to its aggressive nature [4]. Most patients with TSCC are diagnosed with the existing of local invasion or even lymphnode metastasis [5]. Even after active treatments, most patents will still experience recurrence, leading to the poor prognosis [5].

Genetic studies have shown that genetic alterations are frequently observed during the development of TSCC [6, 7]. In effect, genetic approaches have shown promising potentials in the treatment of TSCC [6, 7]. Genome-wide gene expression analysis has also shown that TSCC is also accompanied with changed expression of a huge number of long (> 200 nt) non-coding RNAs (lncRNAs) [8], which are critical determinants in cancer biology [9]. Therefore, in-depth investigations of the function of lncRNAs in TSCC may facilitate its treatment. It has been reported that overexpression of lncRNA CASC15 contributes to the development of colorectal cancer and gastric cancer [10, 11], indicating its oncogenic role. However, the involvement of CASC15 in TSCC is unknown. In addition, prognostic values of lncRNA CASC15 for cancer have not been evaluated by previous studies. Our preliminary deep-sequencing rate revealed that CASC15 was upregulated in TSCC and inversely correlated with miR-214, which has tumor suppressive roles [12]. Our study was carried out to investigate the role of CASC15 in TSCC and explore its interaction with miR-214 as well as the clinical values.

## Materials and methods

### Study design and location

This study is a prospective study that was performed in the city of Jin Zhou, Liaoning Province, China. The Jinzhou population in 2015 comprised 3,068,000 residents.

### Research subjects

Our study included 46 patients with TSCC (30 males and 16 females, 35–66 years, 49.9 ± 5.4 years) who were diagnosed and treated in the first Affiliated Hospital of Jinzhou Medical University between March 2010 and October 2013. Inclusion cirteria:1) newly diagnosed patients; 2) patients willing to participate in 5-year follow-up and signed informed consent; 3) no therapies were initiated before admission. Exclusion criteria: 1) any therapies received before admission; 2) any histories of previous malignancy; 3) patients complicated with other clinical disorders. Ethics Committee of the first Affiliated Hospital of Jinzhou Medical University approved this study (No. 20103548FAHJMU). All clinical examination and lab work were performed by the authors of this paper.

### Tissue specimen collection

All patients were diagnosed by histopathological biopsy. During biopsy TSCC (cancer) and adjacent (within 1 cm around tumors) non-cancer tissues were collected from each patient. Weight of tissue ranged from 0.1 to 0.12 g. All tissue specimens were confirmed by 3 experienced pathologists.

### Treatment and follow-up

Among the 46 patients, 26 received radical resection combined with adjunct chemotherapy or radiation therapy. Other 20 patients only received chemotherapy or radiation therapy, but dose varies according to patients’ conditions. Follow-up study was performed for 5 years or until patients’ deaths. Follow-up was performed through outpatient visit or telephone (every 1–2 months). Patients who were lost during follow-up or died of other caused were excluded from this study. The causes of deaths were determined by reviewing medical record or by the information provided by patients’ families.

### TSCC cell lines

Human TSCC cell lines SCC25 and SCC090 were used in this study to perform all in vitro cell experiments. Cells of both cell lines were bought from ATCC (USA). According to the instructions from ATCC, Dulbecco’s modified Eagle’s medium and Ham’s F12 medium (1:1 mixture) containing 2.5 mM L-glutamine, 0.5 mM sodium pyruvate, 15 mM HEPES, 400 ng/ml hydrocortisone and 10% FBS was used as cell culture medium. Cell culture conditions were 5% CO_2_ and 37 °C. Other details of cell culture were the same as the instructions provided by ATCC.

### RT-qPCR

VWR Life Science Ribozol™ Plus RNA Purification Kit (VWR, USA) was used to extract total RNAs from tissue specimens and SCC25 and SCC090 cells (10^5^ cells). Following reverse transcription performed using AMV Reverse Transcriptase (Promega Corporation, USA), PCR reaction systems were prepared using SYBR Green Master Mix (Bio-Rad, USA) to detect the expression of CASC15 with 18S rRNA as endogenous control. mirPremier™ microRNA Isolation Kit (Sigma-Aldrich, USA) was used to extract miRNAs from tissue specimens and SCC25 and SCC090 cells. Following reverse transcription performed using TaqMan MicroRNA Reverse Transcription Kit (Thermo Fisher Scientific), PCR reaction systems were prepared using TaqMan Real-Time PCR Master Mix (Thermo Fisher Scientific) to detect the expression of miR-124 with U6 as endogenous control. qPCR reactions were repeated 3 times and 2^-ΔΔCT^ method was used to process the data.

### Cell transient transfections

CASC15 expression pcDNA3.1 vector and empty vector were from Sangon (Shanghai, China). Negative control miRNA and miR-124 mimic were from Sigma-Aldrich (USA). SCC25 and SCC090 cells were harvested after overnight culture. All transient cell transfections in to 10^5^ cells were performed using lipofectamine 2000 reagent (Invitrogen, USA) with 10 nM vector and 40 nM miRNA. Cells without transfections were control cells. Cells transfected with negative control miRNA or empty vector were negative control cells. Following experiments were performed at 24 h after transfections.

### Measurement of in vitro cell migration and invasion abilities

SCC25 and SCC090 cells were harvested at 24 h after transfections to prepare single cell suspensions using non-serum cell culture medium. Cell density was adjusted to 3× 10^4^ cells per ml. Cell suspensions were transferred to the upper Transwell chamber (0.1 ml per well), while the lower Transwell chamber was filled with cell culture medium containing 20% FBS. To mimic in vivo cell invasion, upper chamber membranes were coated with Matrigel (356,234, Millipore, USA) at 37 °C for 6 h. Cell invasion and migration were allowed for 3 h, and upper chamber membranes were stained with 0.5% crystal violet (Sigma-Aldrich, USA) for 20 min at room temperature. Stained cells were observed and counted under an optical microscope.

### Statistical analysis

All experiments were repeated 3 times. Differences between TSCC and non-cancer tissues were analyzed by paired t test. Differences among different cell transfection groups were performed by one-way ANOVA and Tukey t test. According to the expression data in TSCC tissues, 46 patients with TSCC were divided into high (*n* = 22) and low (*n* = 24) CASC15 level groups, as well as high (*n* = 21) and low (*n* = 25) miR-124 level groups based on Youden’s index. Correlations between patients’ clinical data and expression levels of CASC15 and miR-124 in TSCC were analyzed by Chi-squared test. Survival curves were plotted using K-M method and compared using log-rank t test. Linear regression was performed to analyze the correlation between expression levels of CASC15 and miR-124. Differences were statistically significant when *p* < 0.05.

## Results

### CASC15 and miR-124 were dysregulated in TSCC tissues

**Fig. 1:**
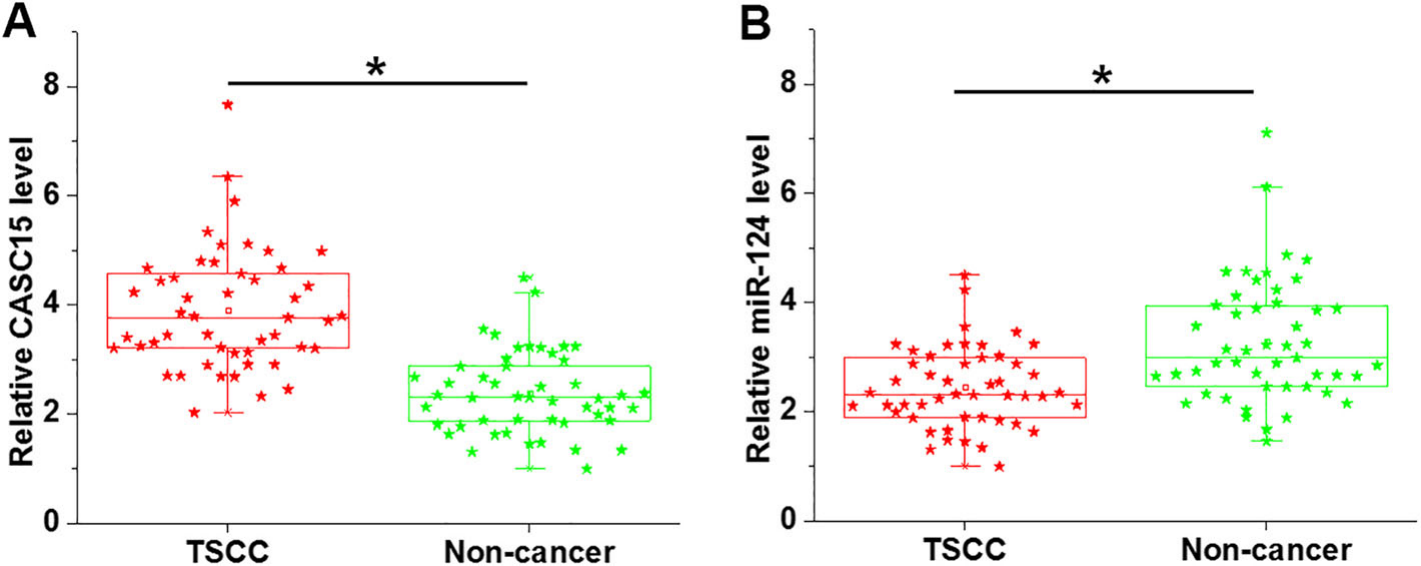
CASC15 and miR-124 were dysregulated in TSCC tissues. Analysis of RT-qPCR data showed that expression of CASC15 was significantly upregulated (**a**), while miR-124 was significantly downregulated (**b**) in TSCC tissues comparing to adjacent non-cancer tissues (*, *p* < 0.05)

CASC15 and miR-124 expression in TSCC tissues and adjacent non-cancer tissues was analyzed by RT-qPCR. Expression data was analyzed by paired t test. Comparing to adjacent non-cancer tissues, expression of CASC15 was significantly upregulated (Fig. 1a, *p* < 0.05), while miR-124 was significantly downregulated (Fig. 1b, *p* < 0.05) in TSCC tissues.

### CASC15 and miR-125 were not affected by clinical stages and predicted survival

**Fig. 2:**
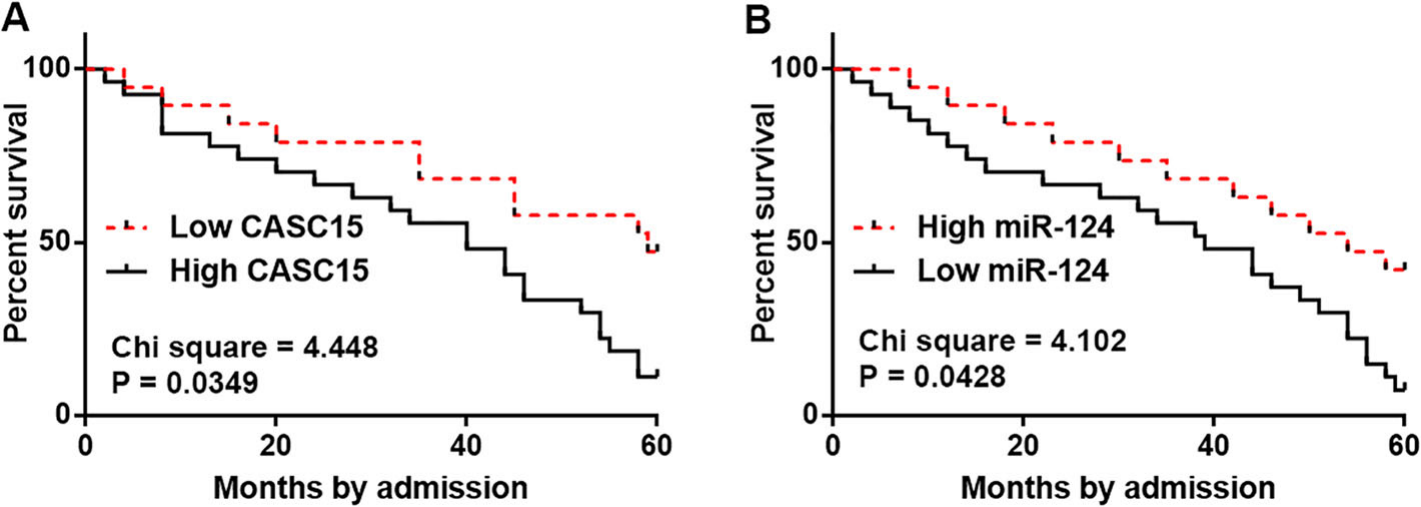
CASC15 and miR-125 were not affected by clinical stages and predicted survival. Analysis of survival curves showed that high CASC15 levels (**a**) and low miR-125 levels (**b**) were significantly correlated with poor survival of TSCC patients

CASC15 and miR-125 expression levels in TSCC tissues were compared among different clinical stages using one-way ANOVA and Tukey t test. No significant differences in CASC15 and miR-125 expression levels were found among patients with different clinical stages (data not shown). According to the expression data in TSCC tissues, 46 patients with TSCC were divided into high (*n* = 22) and low (*n* = 24) CASC15 level groups, as well as high (*n* = 21) and low (*n* = 25) miR-124 level groups based on Youden’s index. Correlations between patients’ clinical data and expression levels of CASC15 and miR-124 in TSCC were analyzed by Chi-squared test. As showed in Tables 1 and 2, expression levels of CASC15 and miR-125 in TSCC were not significantly correlated with patients’ age, gender and clinical stages. Survival curves of both groups were plotted using K-M method and compared using log-rank t test. It was observed that high CASC15 levels (Fig. 2a) and low miR-125 levels (Fig. 2b) were significantly correlated with poor survival of TSCC patients.

**Table 1:** Correlation between patients’ clinical data with CASC15 expression in TSCC tissues

Items	Groups	Cases	High	Low	χ^2^	*p* value
Age (years)	> 50	22	9	13	0.81	0.37
	< 50	24	13	11		
Gender	Male	30	13	17	0.70	0.40
	Female	16	9	7		
AJCC stage	I	8	3	5	2.32	0.51
	II	10	6	4		
	III	17	9	6		
	IV	11	4	7

**Table 2:** Correlation between patients’ clinical data with CASC15 miR-124 expression in TSCC tissues

Items	Groups	Cases	High	Low	χ^2^	*p* value
Age (years)	> 50	22	9	13	0.38	0.54
	< 50	24	12	12		
Gender	Male	30	12	18	1.11	0.29
	Female	16	9	7		
AJCC stage	I	8	3	5	0.57	0.90
	II	10	5	5		
	III	17	8	7		
	IV	11	5	6

### CASC15 and miR-124 were inversely correlated in TSCC

**Fig. 3:**
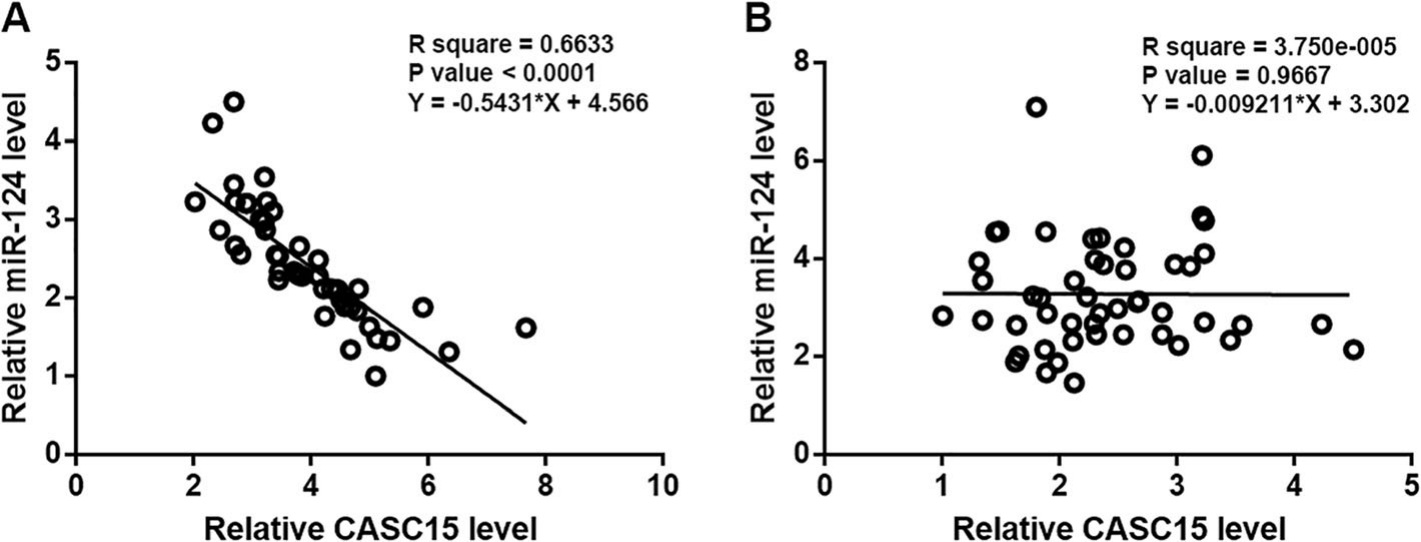
CASC15 and miR-124 were inversely correlated in TSCC. Linear regression showed that expression levels of CASC15 and miR-124 in TSCC tissues were inversely correlated (**a**). But no significant correlation between CASC15 and miR-124 expression level in non-cancer tissues was observed (**b**). Fig. 4 CASC15 overexpression resulted in the downregulation of miR-124. Comparing to two controls (C, control; NC, negative control), expression levels of CASC15 and miR-124 were significantly upregulated at 24 h after transfections (**a**). CASC15 overexpression in TSCC cells caused downregulation of miR-124 (**b**). In contrast, overexpression of miR-124 showed no significant effect on CASC15 expression (*, *p* < 0.05)

Linear regression was performed to analyze the correlation between expression levels of CASC15 and miR-124. It was observed that expression levels of CASC15 and miR-124 in TSCC tissues were inversely correlated (Fig. 3a). However, no significant correlation between CASC15 and miR-124 expression level in non-cancer tissues was observed (Fig. 3b).

### CASC15 overexpression resulted in the downregulation of miR-124

**Fig. 4:**
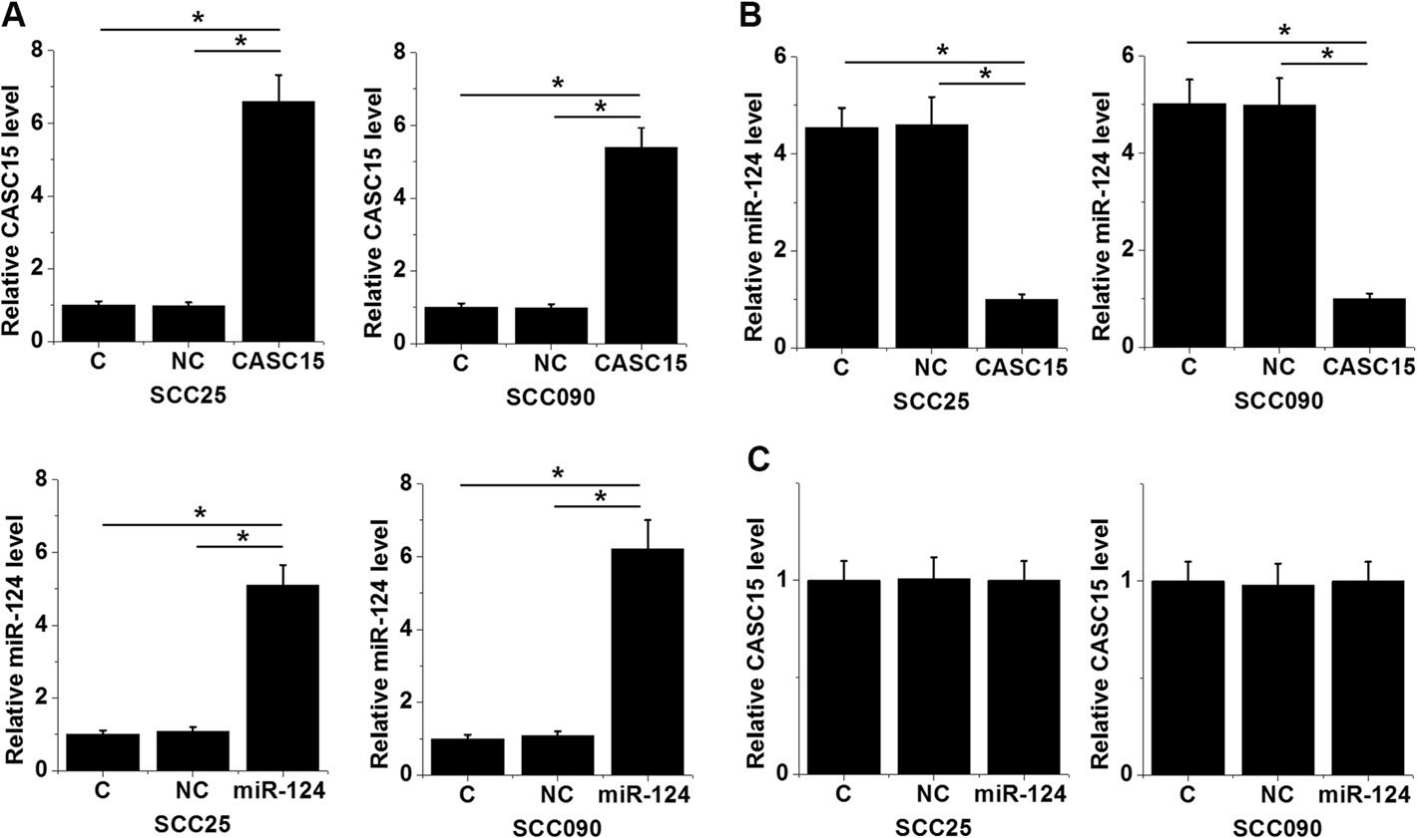
CASC15 regulated TSCC cell migration and invasion through miR-124. Comparing to two controls (**c**, control; NC, negative control), CASC15 overexpression caused the increased, while miR-124 overexpression caused the decreased migration (**a**) and invasion (**b**) rates of TSCC cells. In addition, miR-124 overexpression attenuated the effects of CASC15 overexpression (*, *p* < 0.05)

CASC15 expression vector and miR-124 mimic were transfected into SCC25 and SCC090 cells. Comparing to two controls (C, control; NC, negative control), expression levels of CASC15 and miR-124 were significantly upregulated at 24 h after transfections (Fig. 4a *p* < 0.05). In addition, comparing to two controls, CASC15 overexpression in TSCC cells caused downregulation of miR-124 (Fig. 4b, *p* < 0.05). In contrast, overexpression of miR-124 showed no significant effect on CASC15 expression (Fig. 4c).

### CASC15 regulated TSCC cell migration and invasion through miR-124

**Fig. 5:**
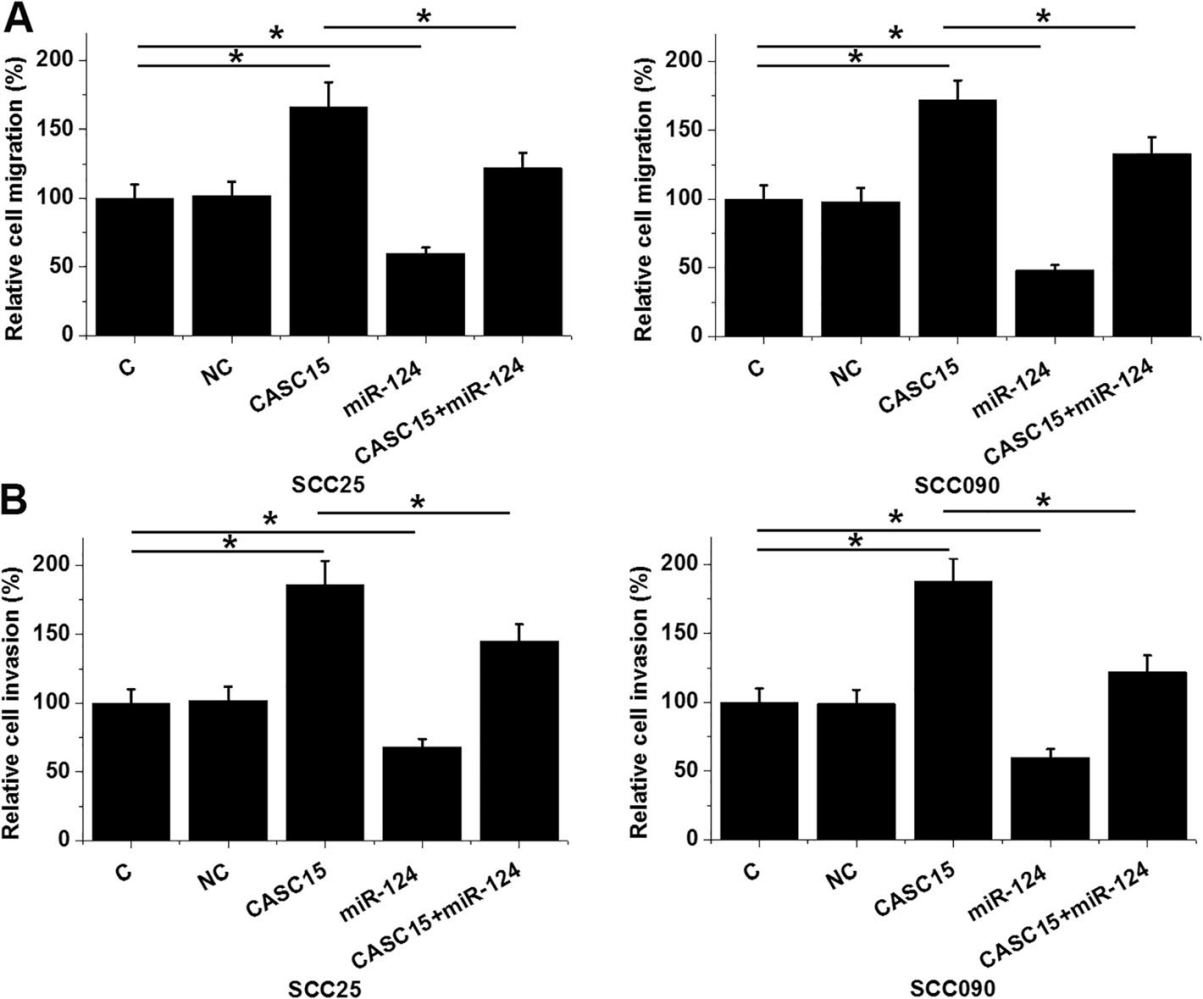
CASC15 regulated TSCC cell migration and invasion through miR-124. Comparing to two controls (C, control; NC, negative control), CASC15 overexpression caused the increased, while miR-124 overexpression caused the decreased migration (**a**) and invasion (**b**) rates of TSCC cells. In addition, miR-124 overexpression attenuated the effects of CASC15 overexpression (*, *p*<0.05)

Transwell migration and invasion assay data were analyzed by one-way ANOVA and Tukey test. Comparing to two controls (C, control; NC, negative control), CASC15 overexpression caused the increased, while miR-124 overexpression caused the decreased migration (Fig. 5a) and invasion (Fig. 5b) rates of TSCC cells (*p* < 0.05). In addition, miR-124 overexpression attenuated the effects of CASC15 overexpression (*p* < 0.05).

## Discussion

Previous studies have shown that CASC15 plays oncogenic roles in the development and progression of colorectal cancer and gastric cancer [10, 11]. Our study first reported that CASC15 was upregulated in TSCC and overexpression of CASC15 may promote TSCC cell migration and invasion by downregulating miR-124, which has tumor suppressive role in TSCC [12].

This study performed a 5-year follow-up study. This is because that the overall survival of TSCC patients is still poor even after active and proper treatment [13]. Overexpression of CASC15 has been observed in colorectal cancer and gastric cancer [10, 11]. The present study also observed the upregulation of CASC15 in TSCC patients. Interestingly, expression of CASC15 was not significantly different among patients with different clinical stages, indicating that this lncRNA may participate in the whole procedure. In addition, patients with high levels of CASC15 in TSCC tissues showed significantly lower overall survival conditions. Therefore, plasma circulating CASC15 may serve as a potential prognostic biomarker for TSCC and facilitate the development of individualized treatment of TSCC. Clinical stages of cancer are closely correlated with survival. CASC15 and miR-125 were not affected by clinical stages. Therefore, the prognostic values of CASC15 and miR-125 were solid. It may also mean that, instead of participating in a specific step of the progression of TSCC, CASC15 and miR-125 may play pivotal roles in the whole process of TSCC. Therefore, analysis of the pre-treatment levels of CASC15 in TSCC patients with proper grouping may assist the prediction of survival time.

Tumor metastasis is the major cause of poor prognosis of TSCC patients [14]. We therefore investigated the effects of CASC15 on TSCC cell migration and invasion. A previously study has shown that CASC15 overexpression resulted in the promoted proliferation of gastric cancer cells [11]. However, our study observed no significant changes in proliferation rates of TSCC cells (data not shown, revealed by CCK-8 assay). However, CASC15 overexpression resulted in increased migration and invasion rates of TSCC cells, indicating that CASC15 can regulate multiple behaviors of cancer cells.

MiR-124 is a well-characterized tumor suppressive miRNA in different types of cancers, such as breast cancer [15], bladder cancer [16] and colon cancer [17]. In a recent study Hunt et al. reported that miR-124 regulated cancer cell motility [12]. Consistently, our study also proved that miR-124 expression has negative effects on the migration and invasion abilities of TSCC cells. In addition, CASC15 may serve as an upstream inhibitor of miR-124 to regulate the migration and invasion of TSCC cells. However, no promising binding site of miR-124 was observed on CASC15. In addition, miR-124 overexpression only partially reduced the effects of CASC15 on cancer cell migration and invasion. Therefore, 1) CASC15 may indirectly affect miR-124 expression; 2) CASC15 may interact with multiple factors to regulate TSCC cell migration and invasion.

Histopathological biopsy is still the gold standard for cancer diagnosis. Therefore detecting the expression of CASC15 in tumor tissues is practical. However, due to the low incidence of TSCC, our study only included 46 patients. Clinical application value of CASC15 still needs further validation by large size clinical trials. Future studies may also focus on the mechanism of the actions of CASC15 in TSCC. It is worth noting that Zuo et al. in a recent study investigated the role of CASC15 in TSCC and they found that CASC15 can target miR-33a-5p to regulate the migration of TSCC cells [18], which is consistent with the data of our studies. However this study also showed that CASC15 overexpression resulted in the accelerated proliferation of TSCC cells, which is inconsistent with our study. This is possibly due to the different cell lines used.

This study failed to perform in vivo animal model experiments and the sample size is small. Our future studies will include animal experiments and enroll more patients to further confirm our conclusions.

## Conclusion

In conclusion, CASC15 was upregulated in TSCC and overexpression of CASC15 may promote TSCC cell migration and invasion by downregulating tumor suppressive miR-124.

## Data Availability

The analyzed data sets generated during the study are available from the corresponding author on reasonable request.

## Abbreviations

lncRNAs: Long (> 200 nt) non-coding RNAs
TSCC: Tongue squamous cell carcinoma
